# Antimicrobial mechanisms and antifungal activity of compounds generated by banana rhizosphere *Pseudomonas aeruginosa* Gxun-2 against *fusarium oxysporum* f. sp. *cubense*

**DOI:** 10.3389/fmicb.2024.1456847

**Published:** 2024-09-25

**Authors:** Junming Lu, Yanbing Huang, Rui Liu, Ying Liang, Hongyan Zhang, Naikun Shen, Dengfeng Yang, Mingguo Jiang

**Affiliations:** ^1^Guangxi Key Laboratory for Polysaccharide Materials and Modifications, School of Marine Sciences and Biotechnology, Guangxi Minzu University, Nanning, China; ^2^Guangxi Key Laboratory of Marine Natural Products and Combinatorial Biosynthesis Chemistry, Guangxi Beibu Gulf Marine Research Center, Guangxi Academy of Sciences, Nanning, China

**Keywords:** *Pseudomonas aeruginosa*, *Fusarium* wilt of banana, antifungal mechanism, phenazine, phenazine-1-carboxylic acid, 2-acetamidophenol, aeruginaldehyde

## Abstract

**Introduction:**

*Fusarium* wilt of banana, also recognized as Panama disease, is caused by the soil-borne fungus *Fusarium oxysporum* f. sp. *cubense* tropical race 4 (FOC TR4). In recent years, strategies utilizing biocontrol agents, comprising antifungal microorganisms and their associated bioactive compounds from various environments, have been implemented to control this destructive disease. Our previous study showed that *Pseudomonas aeruginosa* Gxun-2 had significant antifungal effects against FOC TR4. However, there has been little scientific investigation of the antibacterial or antifungal activity. The aim of this study was to isolate, identify and evaluate the inhibition strength of active compounds in *P. aeruginosa* Gxun-2, so as to explain the mechanism of the strain inhibition on FOC TR4 from the perspective of compounds.

**Methods:**

The main antibacterial compounds of strain Gxun-2 were isolated, purified and identified using by fermentation extraction, silica gel column chromatography, thin-layer chromatography (TLC), high-performance liquid chromatography (HPLC), and nuclear magnetic resonance (NMR) techniques. The effect of the compounds on the mycelial growth, morphology and spore germination of strain FOC TR4 was observed by 96-well plate method and AGAR diffusion method.

**Results:**

Among the metabolites produced by the strain, four antifungal compounds which were identified phenazine (C_12_H_8_N_2_), phenazine-1-carboxylic acid (PCA) (C_13_H_8_N_2_O_2_), 2-acetamidophenol (C_8_H_9_NO_2_) and aeruginaldehyde (C_10_H_7_NO_2_S) were identified through HPLC and NMR. Of these compounds, phenazine and PCA exhibited the most pronounced inhibitory effects on the spore germination and mycelial growth of FOC TR4. Phenazine demonstrated potent antifungal activity against FOC TR4 with a minimum inhibitory concentration (MIC) of 6.25 mg/L. The half-maximal effective concentration (EC_50_) was calculated to be 26.24 mg/L using the toxicity regression equation. PCA exhibited antifungal activity against FOC TR4 with an MIC of 25 mg/L and an EC_50_ of 89.63 mg/L. Furthermore, phenazine and PCA triggered substantial morphological transformations in the mycelia of FOC TR4, encompassing folding, bending, fracturing, and diminished spore formation.

**Discussion:**

These findings indicate that strain Gxun-2 plays a crucial role in controlling FOC TR4 pathogenesis, predominantly through producing the antifungal compounds phenazine and PCA, and possesses potential as a cost-efficient and sustainable biocontrol agent against *Fusarium* wilt of banana in forthcoming times.

## Introduction

Banana (*Musa* spp.), is one of the most important fruit crops, extensively cultivated and consumed globally (Justine et al., 2022; Yang et al., 2021). Nonetheless, the worldwide banana industry has incurred substantial economic losses owing to the rampant spread of *Fusarium* wilt of banana (Dale et al., 2017; Ismaila et al., 2023). *Fusarium* wilt of banana is a destructive soil-borne fungal disease caused by *Fusarium oxysporum* f. sp. *Cubense* with the tropical race 4 (FOC TR4) causing the most severe damage among the four races of this pathogen (Pegg et al., 2019; Zhan et al., 2022). The pathogen infiltrates the xylem tissue of the root, propagating upward via the vascular system of the pseudostem, thereby impeding the banana’s water and nutrient absorption capabilities and disrupting its typical growth and development (Li et al., 2017; Warman and Aitken, 2018). Furthermore, fusaric acid (FSA) and various toxins are exuded by FOC TR4 within the rhizosphere, profoundly impacting the roots, stems, leaves, and fruits of banana plants, inducing pathological responses like tissue decay and necrosis, culminating in banana wilt and eventual demise (López-Díaz et al., 2018). The eradication of FOC TR4 upon its introduction or detection in banana orchards proves exceedingly challenging (Roberts et al., 2024; Zhang et al., 2024). This is attributed to the enduring survival of FOC TR4 as chlamydospores or as a saprophyte of non-host plants in soil (Shen et al., 2019; Were et al., 2023). *Fusarium* wilt of banana is notably infectious and detrimental, emerging as a primary impediment to global banana cultivation (Fan et al., 2021; Fatin et al., 2020). Therefore, it is urgent to effectively control the occurrence and distribution of FOC TR4 in banana.

Despite the potential of physical isolation and chemical fungicides to mitigate the dissemination of *Fusarium* wilt and enhance banana yield, these approaches are marred by inefficiency and induce secondary environmental pollution (Li et al., 2022; Siamak and Zheng, 2018). Biological control can effectively control *Fusarium* wilt of banana and inhibit the growth of pathogenic bacteria by using microorganisms or secondary metabolites (Pushpavathi et al., 2016; Zhu et al., 2023). *Bacillus* and *Pseudomonas* strains have undergone extensive investigation and application in suppressing FOC TR4 owing to their direct or indirect advantageous attributes (Dimkić et al., 2022). For example, *Bacillus amyloliquefaciens* S185 exhibited robust antifungal efficacy (78%) against FOC TR4 and the antifungal compound iturin A5 was isolated and identified (Singh et al., 2021). Additionally, *Burkholderia* HQB-1 from the rhizosphere soil of bananas, had good antibacterial activity against FOC TR4, mainly through the production of the antifungal compound phenazin-1-carboxylic acid (PCA) (Xu et al., 2020). *Pseudomonas*, exhibiting remarkable diversity, thrives in diverse natural environments including soil, water, plant and animal hosts, and various biological and environmental niches (Khatri et al., 2023; Silby et al., 2011). Typical *Pseudomonas* species encompass *Pseudomonas fluorescens, Pseudomonas aeruginosa, Pseudomonas lororaphis, Pseudomonas aureofaciens* among others (Silby et al., 2011). *Pseudomonas* and its secondary metabolites exert significant control over plant pathogenic fungi (Dimkić et al., 2022; Götze and Stallforth, 2020). For example, *P. fluorescens* 2–79 and *P. aureofaciens* 30–84, which produced the antibiotic phenazine-1-carboxylic acid (PCA) and effectively suppress take-all disease of wheat, a significant root disease of wheat caused by *Gaeumannomyces graminis* var. *tritici* (Rieusset et al., 2020; Thomashow et al., 1990). *P. fluorescens* Pf-5 and CHA0 produced Pyoluteorin (Plt), Pyrrolnitrin (Prn) and 2,4-diacetylphloroglucinol (2,4-DAPG), which inhibited root rot and cataplakia (Nowak-Thompson et al., 1999; Schnider et al., 1995).

Over recent years, a succession of *P. aeruginosa* strains originating from rhizosphere soil, exhibiting potent biological control efficacy, have been meticulously isolated and characterized (Shanmugaiah et al., 2010; Zhou et al., 2016). The secondary metabolites of these *P. aeruginosa* strains exhibit a rich diversity, effectively combating a spectrum of plant fungal or bacterial diseases (Neve et al., 2024; Xie et al., 2023). Simultaneously, these metabolites play a pivotal role in bolstering their ecological adaptability and ensuring successful reproduction and survival within the intricate rhizosphere milieu (Xie et al., 2023). For example, *P. aeruginosa* PNA1 exhibited potent suppression against *Fusarium* wilt of chickpea and *Pythium* damping-off of bean through producing the phenazine antibiotics PCA and Oxychloraphine (Anjaiah et al., 1998). And, *P. aeruginosa* ID 4365 synthesized siderophores and PCA, effectively impeding the growth of *Sclerotium rolfsii*, *Aspergillus niger* and various other phytopathogens (Rane et al., 2008). Nevertheless, numerous novel natural compounds synthesized by *P. aeruginosa* remain unexplored (Pratiwi et al., 2017). The processes of isolating, purifying, and identifying these bioactive natural products, particularly those with antimicrobial properties, warrant comprehensive investigation.

Therefore, the objective of the present research was to determine antifungal activity of compounds generated by *P. aeruginosa* Gxun-2 and delineate their antimicrobial mechanism against FOC TR4. The main antibacterial compounds of strain Gxun-2 were isolated, purified and identified using by fermentation extraction, silica gel column chromatography, thin-layer chromatography, high-performance liquid chromatography (HPLC), and nuclear magnetic resonance (NMR) techniques. The possible mechanism of antifungal activity was studied by investigating the effects of the compounds on mycelial growth, morphology and the spore germination of FOC TR4. The aim of this study was to analyze the antifungal metabolites from potential beneficial microbial resources and to identify the main efficient compound that could be used to prevent *Fusarium* wilt of banana.

## Materials and methods

*P. aeruginosa* Gxun-2 was isolated from the rhizosphere of healthy banana in Guangxi province, China (Li et al., 2022). It is preserved in the Guangdong Microbial Culture Collection Center (GDMCC No.61615). FOC TR4 were obtained from the Guangxi Academy of Agricultural Sciences, Nanning, China. They were cultured on potato dextrose agar (PDA) medium at 30°C for 7 d. Phenazine and phenazine-1-carboxylic acid (PCA) are standard products purchased from Shanghai yuanye Bio-Technology Co., Ltd. The Agilent TC-C18 column (5 μm, 250 × 4.6 mm) was used to detect isolated components on the Waters E2695 HPLC instrument. The optimized culture medium and culture conditions were as follows: sodium succinate 2 g/L, glycerin 10 g/L, ammonium sulfate 1 g/L, pH was adjusted to 7.2, 35°C, 200 rpm.

### Inhibition of *Pseudomonas aeruginosa* Gxun-2 extracts to FOC TR4

The inhibitory impact of strain Gxun-2 extracts on the growth of FOC TR4 mycelia was investigated using a modified version of the previously established method (Ji et al., 2018). A fungal disc of FOC TR4, with a diameter of 7.5 mm, was centrally placed on a PDA plate. Uniformly spaced 7.5 mm diameter holes were drilled at four equidistant points, each approximately 2.5 cm from the plate center. Subsequently, 100 μL of Gxun-2 extracts were inoculated into each hole. Concurrently, 100 μL of ultrapure water were inoculated into each hole as the control. After a 5-day incubation period at 28°C, the inhibition zone was defined by measuring the distance between the periphery of the fungal mycelium and the aperture where the crude extract was introduced. All strains were subjected to triplicate experimental trials. The inhibitory effects of antagonistic strains on pathogenic bacteria were observed, followed by calculating the inhibition rate. Additionally, the morphology of mycelia at the periphery of bacterial colonies was analyzed using scanning electron microscopy (SEM). Mycelial radial inhibition rate = *Σ* (average diameter of target colony in control group-average diameter of target colony in experimental group)/average diameter of target colony in control group *×* 100% (Zhang et al., 2022).

The inhibition effect of spore germination of FOC TR4 by Gxun-2 extracts was examined using a modified version of the previously established method (Wang et al., 2023). FOC TR4 mycelia cultured on PDA solid medium were transferred to PDA liquid medium and incubated at 28°C and 200 rpm for 3 days. Following filtration through 8 layers of gauze to remove mycelia, the spore concentration was adjusted to 5 × 10^5^ CFU/mL using a hemocytometer and stored at 4°C for subsequent experiments. Once the sterilized PDA was cooled to 50°C, the spore suspension of FOC TR4 was added at the dosage of 5%, and the plate was turned upside down after being thoroughly mixed. Upon solidification, holes were drilled using a hole punch in two perpendicular directions, 2.5 cm from the plate center. 100 μL of Gxun-2 extracts were added to each hole, with methanol serving as the control. Each group was tested in triplicate. The bacteriostatic effect was observed after a 5-day incubation at 28°C.

### Culture and extraction of antibacterial compounds

*P. aeruginosa* Gxun-2 was cultivated on Luria-Bertani (LB) solid medium at 35°C for a duration of 7 days. Single colonies were meticulously chosen and introduced into a 1 L conical flask containing 250 mL of LB liquid medium, incubated at 35°C and 200 rpm for a duration of 3 days to initiate the formation of a seed suspension. In order to acquire a substantial volume of culture broth, 100 mL of the seed culture both was inoculated in 40 flasks (1 L) containing 250 mL of optimized iron-enriched medium. The conical flasks were incubated at 35°C with agitation at 200 rpm for 3 days. This process was repeated 7 times to yield a total volume of 70 L of fermentation broth. Subsequently, the resulting fermentation mixture underwent centrifugation at 8,000×*g* for a duration of 20 min to facilitate the separation of the supernatant and cellular precipitate (comprising nutrient medium), following which the supernatant was meticulously collected. Following this, 1.5 times the volume of ethyl acetate (EA) was introduced into the supernatant, gently agitated, and allowed to stand for a period of 4 h prior to the careful extraction of the EA layer. This procedure was repeated three times. The EA layers were combined and the solvent was evaporated under vacuum at 42°C using a rotary evaporator (EYELA, N-1300, Japan), yielding a fermentation extract weighing 26.67 g. The Gxun-2 extract was stored at 4°C.

### Isolation of antibacterial substances and determination of antibacterial activity

The 26.67 g of fermentation extract were dissolved in dichloromethane and subsequently filtered through filter paper to remove any impurities. Following the thorough mixing of 20 g of silica gel powder into the sample, the mixture was loaded onto the silica gel column. The crude extracts were eluted using a linear gradient (1 L each) of dichloromethane: methanol (100: 0, 100: 1, 100: 2, 100: 3, 100: 4, 100: 5, 100: 6, 100: 7, 100: 9, 100: 10, 100: 11, 100: 12, 100: 13, 100: 15, 100: 16, 100: 17, 100: 18, 100: 19, 100: 20, 100: 21, 100: 22, 100: 23, 100: 24, 100: 25, 100: 50, 0: 100) on a silica gel column (8.0 cm inner diameter, 60 cm length) and fractionated into 27 fractions. Subsequently, the 27 fractions were scrutinized and amalgamated utilizing thin layer chromatography (TLC) and HPLC, subsequently categorized and consolidated into 7 distinct fractions denoted as Fr1 to Fr7 (Jing et al., 2020).

The antimicrobial efficacy of the seven fractions (Fr1-Fr7) against FOC TR4 was assessed using a previously established method with minor adjustments, aimed at identifying the locations of the principal antimicrobial compounds (Li et al., 2021). Four plates of PDA medium were prepared, and holes were punctured at the center of each plate using a hole punch. Subsequently, 100 microliters of Fr1-Fr7 fractions were sequentially added to the wells, followed by an equal volume of methanol as a control. FOC TR4 fungal discs with a diameter of 7.5 mm were inoculated in the center of each plate. Three replicates were set for each group. The incubator was maintained at 28°C for 24 h, followed by inversion and further incubation for an additional 5 days. Following the experiment investigating the bacteriostatic activity of FOC TR4, the most potent fraction (Fr2) showing visible inhibition was chosen for further isolation. Fr2 (3.09 g) was applied to a silica gel column (GF 254, 3 × 50 cm, Qingdao Marine Chemical Factory, China) wet with chloroform. Using a linear gradient (1 L each) of chloroform: methanol system (100: 0; 95: 5; 90: 10; 80: 20; 70: 30; 50: 50) were utilized for gradient elution, followed by HPLC and TLC to obtain subfractions Fr2-L1, Fr2-L2, Fr2-L3.

### TLC and HPLC analysis

Subsequent to elution, the 27 fractions of the crude extract were amalgamated through TLC and HPLC analysis. The specimen was applied onto a 0.25 mm silica gel TLC plate and underwent development in a solvent system comprising trichloromethane and methanol (in a ratio of 15: 1). The dried and developed TLC plates were scrutinized under UV radiation at a wavelength of 254 nm, following which they were positioned within an ultraviolet chamber. A total of 27 separation stages were identified via HPLC analysis. An Agilent TC-C18 column (5 μm, 250 × 4.6 mm) was employed in conjunction with the Waters E2695 HPLC instrument for the elution of mobile phases A and B, with 0.1% glacial acetic acid and acetonitrile with 0.1% glacial acetic acid serving as the mobile phases. The detection wavelength of the Diode Array Detector (DAD) was set at 248 nm, while the flow rate was maintained at 1.0 mL/min. A sample volume of 20 μL was utilized. The gradient elution program utilized in this study is delineated as follows: (aqueous solution containing 0.1% glacial acetic acid (A) and acetonitrile solution containing 0.1% glacial acetic acid (B)) from 0 to 5 min, 10% B; from 5 to 8 min, 40% B; from 8 to 10 min, 80% B; from 10 to 13 min, 100% B; from 13 to 18 min, 10% B; from 18 to 20 min, 0% B; from 20 to 25 min, 0% B. The post-run duration was established as 5 min. The post-run duration was established as 5 min.

### Purification and identification of the main antifungal substances

For the isolation of highly pure compounds, Fr2-L1and Fr2-L2 were all separated by HPLC in a reversed-phase C18 column Agilent TC-C18 column (5 μm, 250 × 4.6 mm) in high performance liquid chromatography (HPLC, Waters). Fr2-L1 was water with 0.1% acetic acid (v/v)/ acetonitrile with 0.1% acetic acid system [0–15 min: 50%, compound 1 (20 mg, t_R_ = 4.98 min), compound 2 (15 mg, t_R_ = 7.28 min) and compound 4 (10 mg, t_R_ = 8.78 min) were obtained at the flow rate of 1 mL/min]. Compound 3 (16.6 mg, t_R_ = 4.82 min) was isolated from Fr2-L2 (35% water with 0.1% acetic acid (v/v)/ acetonitrile with 0.1% acetic acid iso-degree elution for 20 min, flow rate 1 mL/min). Subsequently, the structure of each compound was elucidated through spectroscopic analysis. Four compounds were solved in deuterated chloroform (CDCl_3_) at the concentration of 10 mg/mL for the ^1^H-NMR by an Agilent 800 MHz DD2 spectrometers (Agilent, United States). The NMR spectra were processed and analyzed using MestReNova software (version 6.1.16384).

### Inhibitory effects of phenazine, PCA and 2-acetamidophenol on FOC TR4 spore germination

The antifungal activity of the pure compound isolated from *P. aeruginosa* Gxun-2 was assessed using a standardized 96-well microdilution broth assay (Wedge and Kuhajek, 1998). The minimum inhibitory concentrations (MICs) of phenazine, PCA and 2-acetamidophenol from the culture broth of strain Gxun-2 against FOC TR4 were determined. Phenazine, PCA and 2-acetamidophenol were subjected to serial two-fold dilutions to achieve various concentrations (800.00, 400.00, 200.00, 100.00, 50.00, 25.00, 12.50, 6.25, 3.13, 1.56, 0.78, 0.39 and 0.20 mg/L) for the MIC assay. The MIC was defined as the lowest concentration of PCA inhibiting fungal growth. Carbendazim served as the positive control, while 0.1% DMSO and methanol served as the negative control. After incubation at 28°C for 24 h, spore germination was observed under an inverted microscope. All experiments were conducted in triplicate (Xu et al., 2020). For each treatment group, 100 spores were counted, and the spore germination inhibition rate was calculated using the following formula (Wei et al., 2017).


$$\mathrm{Spore germination rate}\;\left(\%\right)=\frac{\mathrm{Spore count number}}{100}\times 100\%$$

### Growth inhibition of FOC TR4 mycelial growth by phenazine, PCA and 2-acetamidophenol

The antimicrobial activity was assessed employing an agar-well diffusion method (Wonglom et al., 2019). Phenazine was added to autoclaved PDA medium and subsequently diluted to different concentrations of 0, 0.78, 1.56, 3.12, 6.25, 12.50, 25, 50, 100 and 200 mg/L, respectively. PCA was introduced into autoclaved PDA medium and subsequently diluted to different concentrations of 0, 1.56, 3.12, 6.25, 12.5, 25, 50, 100, 200 and 400 mg/L, respectively. 2-Acetamidophenol was introduced into autoclaved PDA medium and subsequently diluted to different concentrations of 0, 25, 50, 100, 200, 400, 800, 1,600, 3,200 and 6,400 mg/L, respectively. Consistent concentrations of methanol and DMSO were used as negative controls in each group. A 7.50-mm-diameter fungal disc of FOC TR4 was inoculated in the center of plate. The diameter of FOC TR4 growth was measured after incubating for 5 days at 28°C. Each treatment was replicated three times. The least squares method was employed to formulate a linear regression equation, specifically a toxicity regression equation (Van Ewijk and Hoekstra, 1993). The half-maximal effective concentration (EC_50_) value was determined based on the toxicity regression equation.

### Effects of phenazine, PCA and 2-acetamidophenol on the mycelial morphological characteristics of FOC TR4

Phenazine, PCA, and 2-acetamidophenol were individually added to autoclaved PDA medium and diluted to a final concentration of 200 mg/L. Subsequently, fungal discs of FOC TR4, each with a diameter of 7.50 mm, were inoculated onto PDA plates. Sterilized cover slips were positioned adjacent to the fungal discs. Following a 5-day incubation at 28°C, the cover slips were removed and transferred to sterile petri dishes. The mycelial morphology of FOC TR4 was observed using the DM2000 LE microscope (Leica, Germany) (Yun et al., 2022).

### Statistical analysis

Each experiment was independently conducted a minimum of three times. The data were analyzed using GraphPad Prism version 8. Statistical analysis was conducted using IBM SPSS statistics 26. Differences among treatments were assessed using one-way analysis of variance (ANOVA), followed by Duncan’s multiple range test on the means, with statistical significance defined as *p* < 0.05. A *p*-value of less than 0.05 or 0.01 was deemed statistically significant.

## Results

### Impact of strain Gxun-2 extracts on FOC TR4 mycelium growth and spore germination

**Figure 1:**
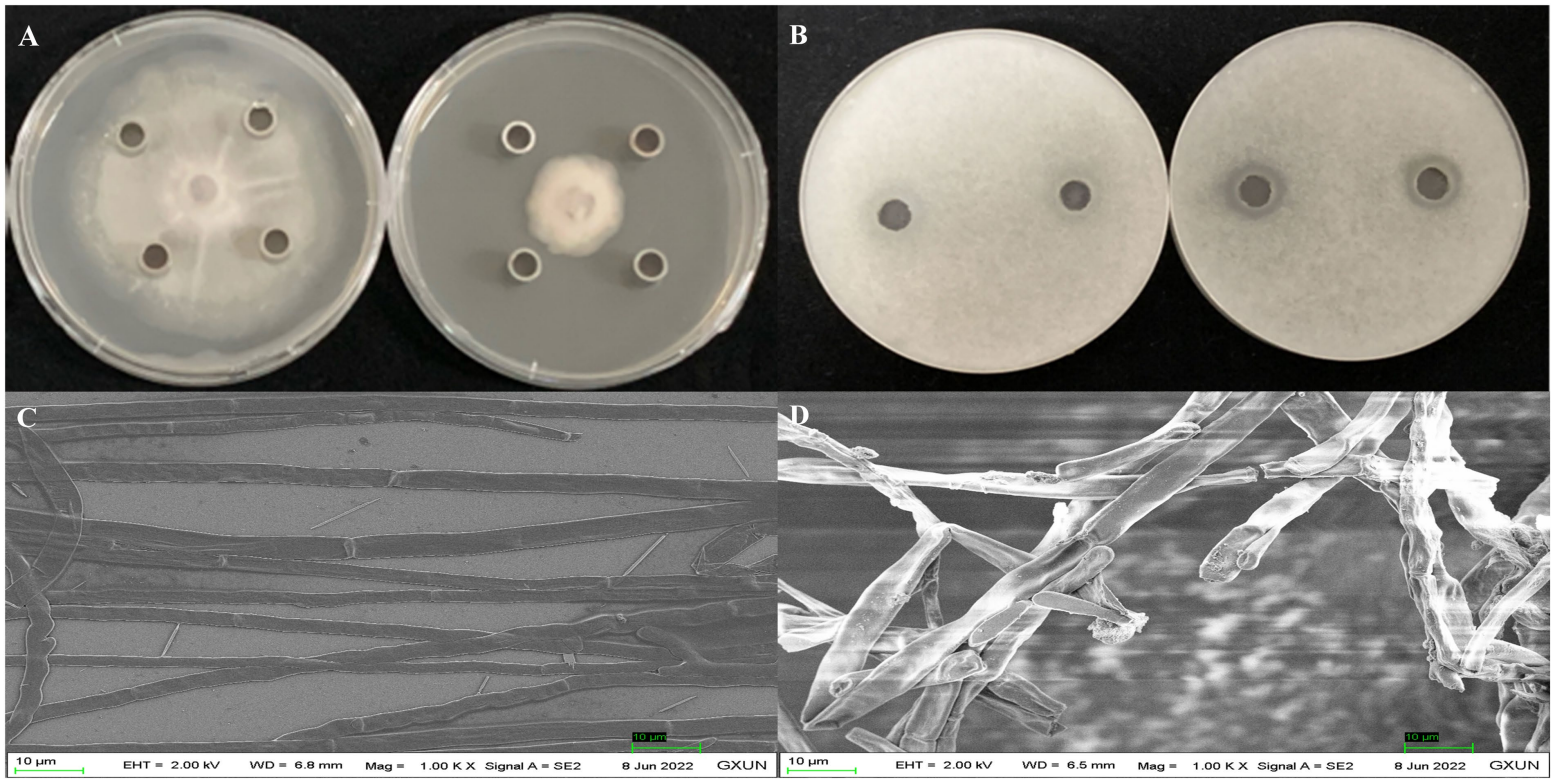
Antagonistic effects of strain Gxun-2 extracts against FOC TR4. **(A)** Inhibition of FOC TR4 mycelial growth by Gxun-2 extracts; **(B)** Suppression of FOC TR4 spore germination by Gxun-2 extracts; **(C)** Normal morphology of FOC TR4 mycelium under scanning electron microscope; **(D)** Effect of Gxun-2 extract on the morphology of FOC TR4 mycelium.

After 5 days of incubation at 28°C, the supernatant extracts from strain Gxun-2 demonstrated substantial inhibition of FOC TR4 growth, achieving inhibition rates occasionally as high as 65.55 ± 0.65%. The appearance of a clear zone upon the application of crude extracts provided evidence of their inhibitory effect on FOC TR4 germination. Scanning electron microscopy (SEM) revealed vigorous growth of FOC TR4 mycelia in the absence of strain Gxun-2 extracts, characterized by uniform thickness and smooth texture. In contrast, mycelia in the group treated with FOC TR4 and strain Gxun-2 extracts displayed distortions, thickening, increased branching, disordered growth, curvature, and knotting (Figure 1). The results showed that the extracts of strain Gxun-2 could inhibit the normal growth of FOC TR4 and change the mycelial morphology.

### Separation and purification of main antibacterial active substances

**Figure 2:**
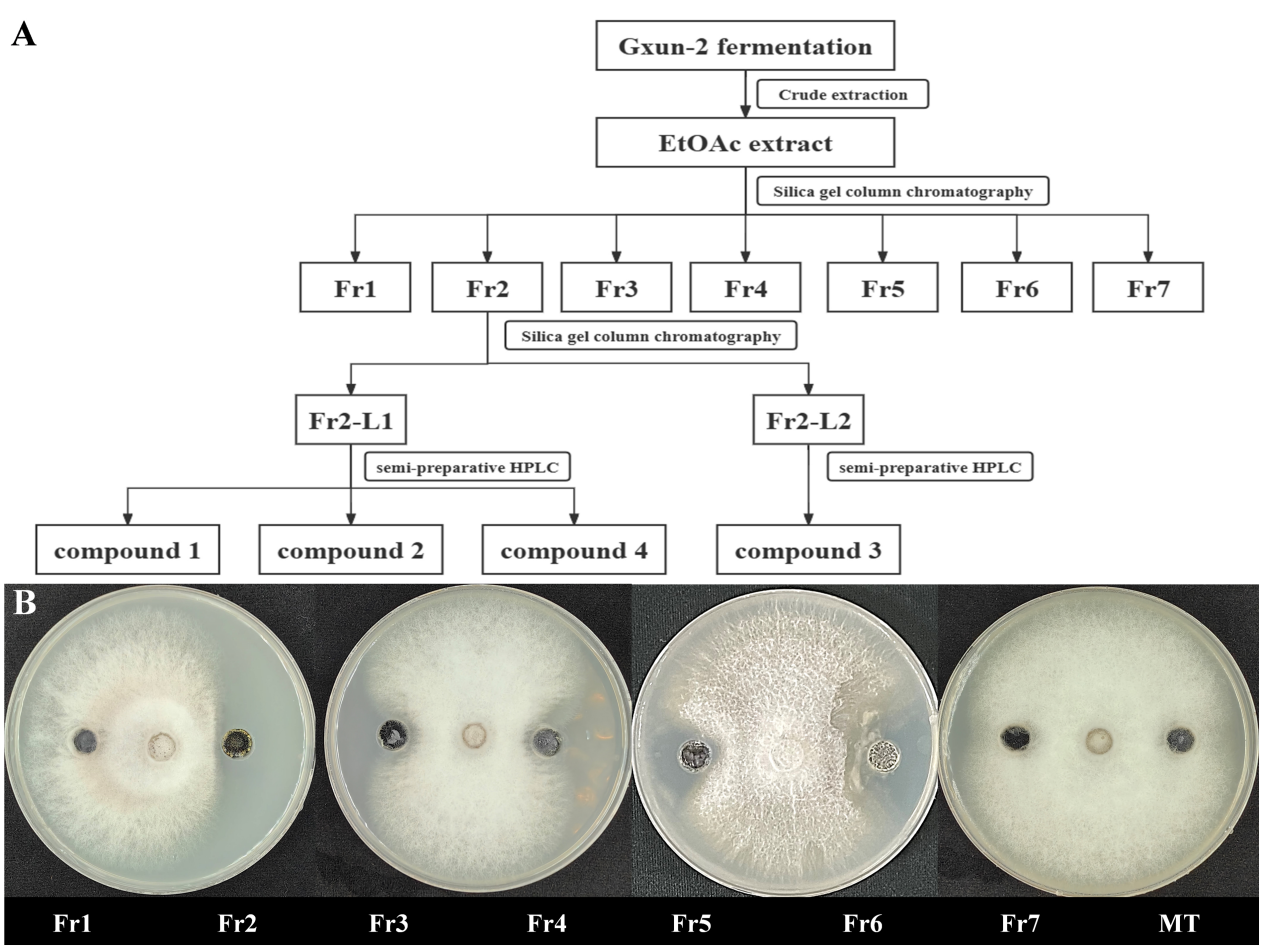
Isolation of main active compounds of strain Gxun-2. **(A)** Flow chart showing extraction and separation procedure of compounds from strain Gxun-2; **(B)** The activity experiment of Fr1, Fr2, Fr3, Fr4, Fr5, Fr6, Fr7, MT on FOC TR4.

Strain Gxun-2 demonstrates robust antagonistic activity against FOC TR4. To isolate and purify its primary active constituents, the fermentation supernatant of strain Gxun-2 was roughly extracted, resulting in the acquisition of 26.67 g of ethyl acetate extract. Assisted by activity tracing, Fr2 demonstrates remarkable antibacterial efficacy, with Fr7 exhibiting a subsequent effect. Fr2 underwent analysis via HPLC and TLC, and the purity of the compounds in Fr2 was relatively high, and the content of primary antibacterial substances was higher. Following this, Fr2 underwent additional separation via silica gel column chromatography to yield Fr2-L1and Fr2-L12. From these fractions, individual compounds were isolated using high-performance liquid chromatography, resulting in the isolation of four compounds (Figure 2).

### Identification of the structure of antibacterial substances

**Figure 3:**
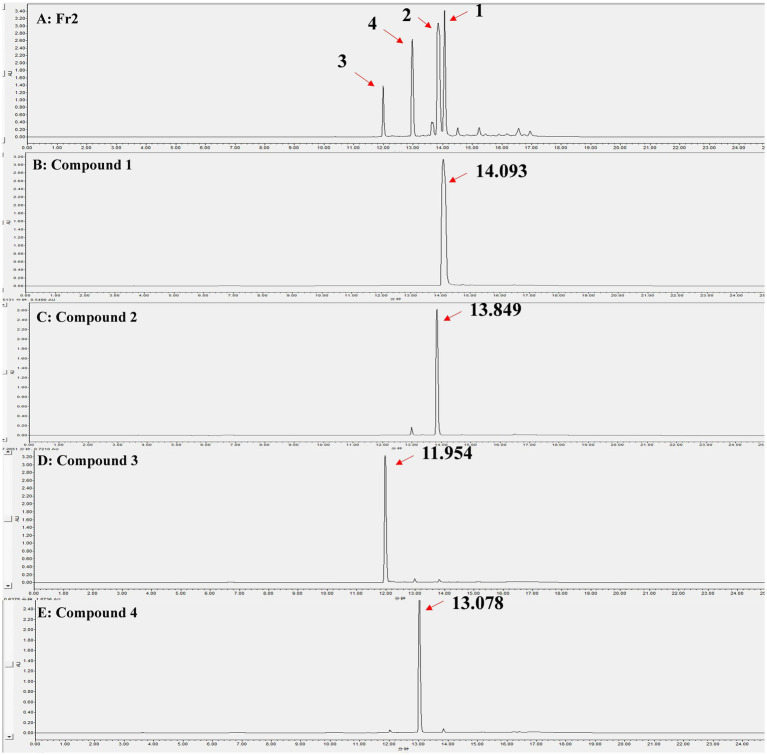
HPLC analysis of the Fr2 and 4 compounds, the peaks indicated by the direction of the red arrows are the isolated compounds 1–4 and their retention times. **(A)** Fr2; **(B)** Compound 1; **(C)** Compound 2; **(D)** Compound 3; **(E)** Compound 4.

**Figure 4:**
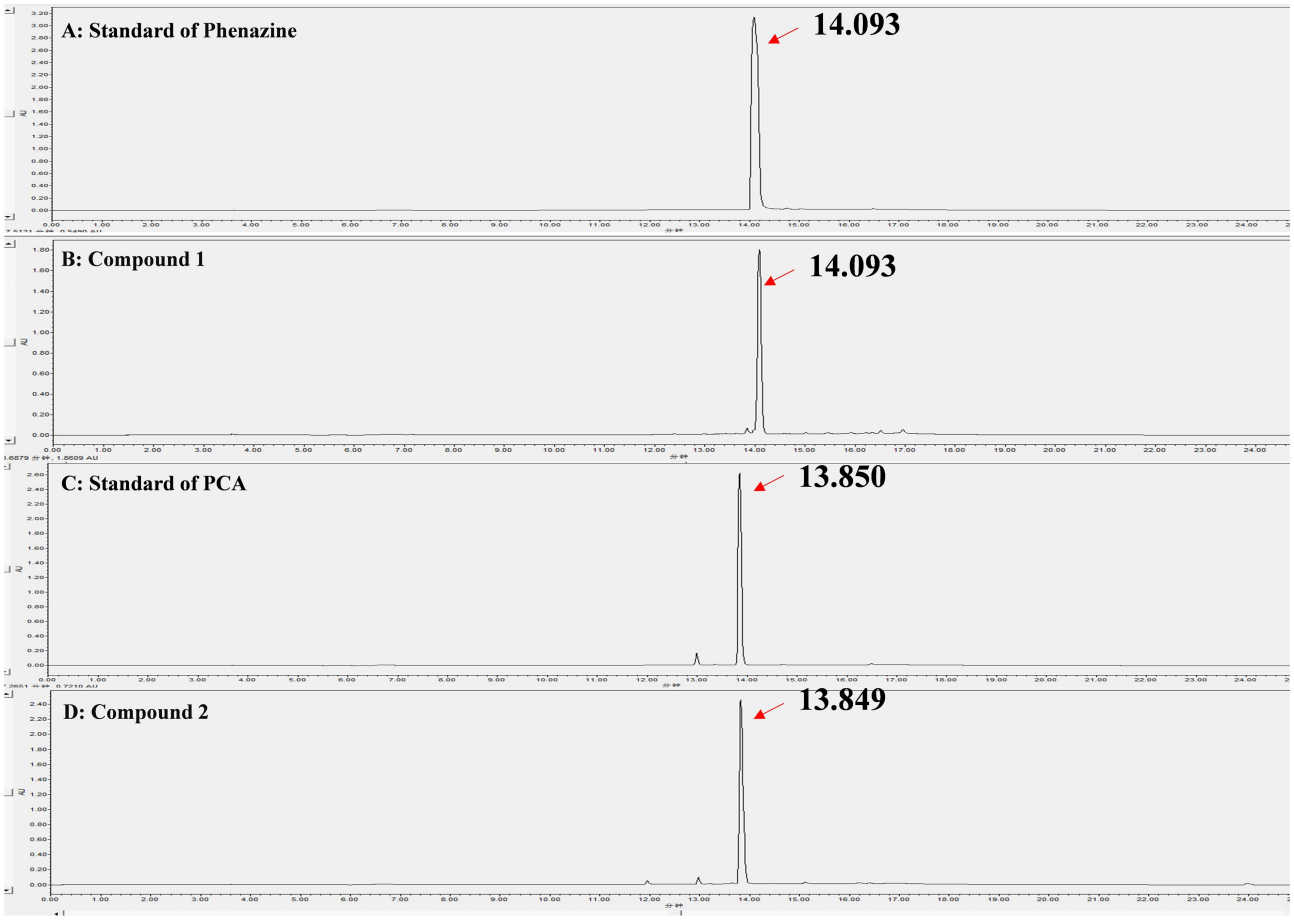
Identification of the compound 1 and compound 2 by HPLC. **(A)** Standard of phenazine; **(B)** Compound 1; **(C)** Standard of PCA; **(D)** Compound 2.

Through liquid phase analysis, the four compounds we obtained were the main compounds of Fr2. The retention times of compounds 1–4 were 14.093, 13.849, 11.954 and 13.078 min, severally (Figure 3). Comparison of retention times between standards and compounds via HPLC revealed that compound 1 corresponded to phenazine due to its retention time, while compound 2 closely matched PCA. This enabled accurate identification of phenazine as compound 1 and PCA as compound 2 (Figure 4).

**Figure 5:**
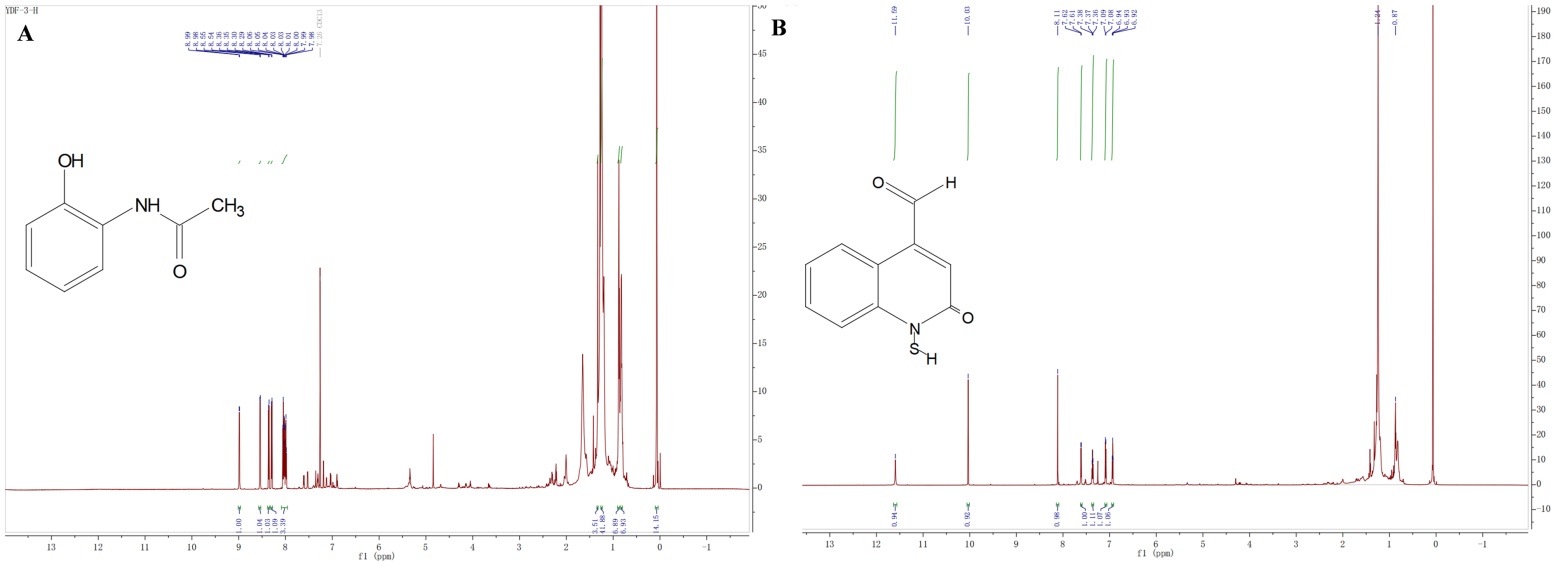
**(A)** Hydrogen spectrograms and structural formula of compounds 3; **(B)** Hydrogen spectrograms and structural formula of compounds 4.

The compound 3: white solid. ^1^H NMR (800 MHz, Chloroform-*d*) *δ* 8.99 (d, *J* = 7.0 Hz, 1H), 8.54 (d, *J* = 8.8 Hz, 1H), 8.36 (d, *J* = 8.7 Hz, 1H), 8.29 (d, *J* = 8.6 Hz, 1H), 8.09–7.96 (m, 3H), 1.33 (s, 4H), 1.26–1.23 (m, 42H), 0.87 (t, *J* = 7.1 Hz, 7H), 0.84–0.80 (m, 7H), 0.07 (s, 14H). All these data were compared with published NMR data and were consistent with the data for 2-acetamidophenol (Figure 5A). The compound 4: white solid, ^1^H NMR (800 MHz, Chloroform-*d*) δ 11.59 (s, 1H), 10.03 (s, 1H), 8.11 (s, 1H), 7.61 (d, *J* = 7.9 Hz, 1H), 7.39–7.34 (m, 1H), 7.09 (d, *J* = 8.3 Hz, 1H), 6.93 (t, *J* = 7.5 Hz, 1H). All these data were compared with published NMR data and were consistent with the data for N-mercapto-4-formylcarbostyril (Figure 5B) (Fakhouri et al., 2001). Structure revision of N-mercapto-4-formylcarbostyril produced by *P. fluorescens* G308 to 2-(2-hydroxyphenyl)-thiazole-4-carbaldehyde [aeruginaldehyde] (Ye et al., 2014). Aeruginaldehyde, a by-product of the siderophore pyochelin biosynthesis or degradation and that the ambABCDE genes are not responsible for IQS (2-(2-hydroxylphenyl)-thiazole-4-carbaldehyde) synthesis (Cornelis, 2020). The amount of aeruginaldehyde isolated in this experiment is insufficient to investigate its antibacterial activity against FOC TR4.

### Phenazine, PCA and 2-acetamidophenol inhibited spore germination of FOC TR4

**Figure 6:**
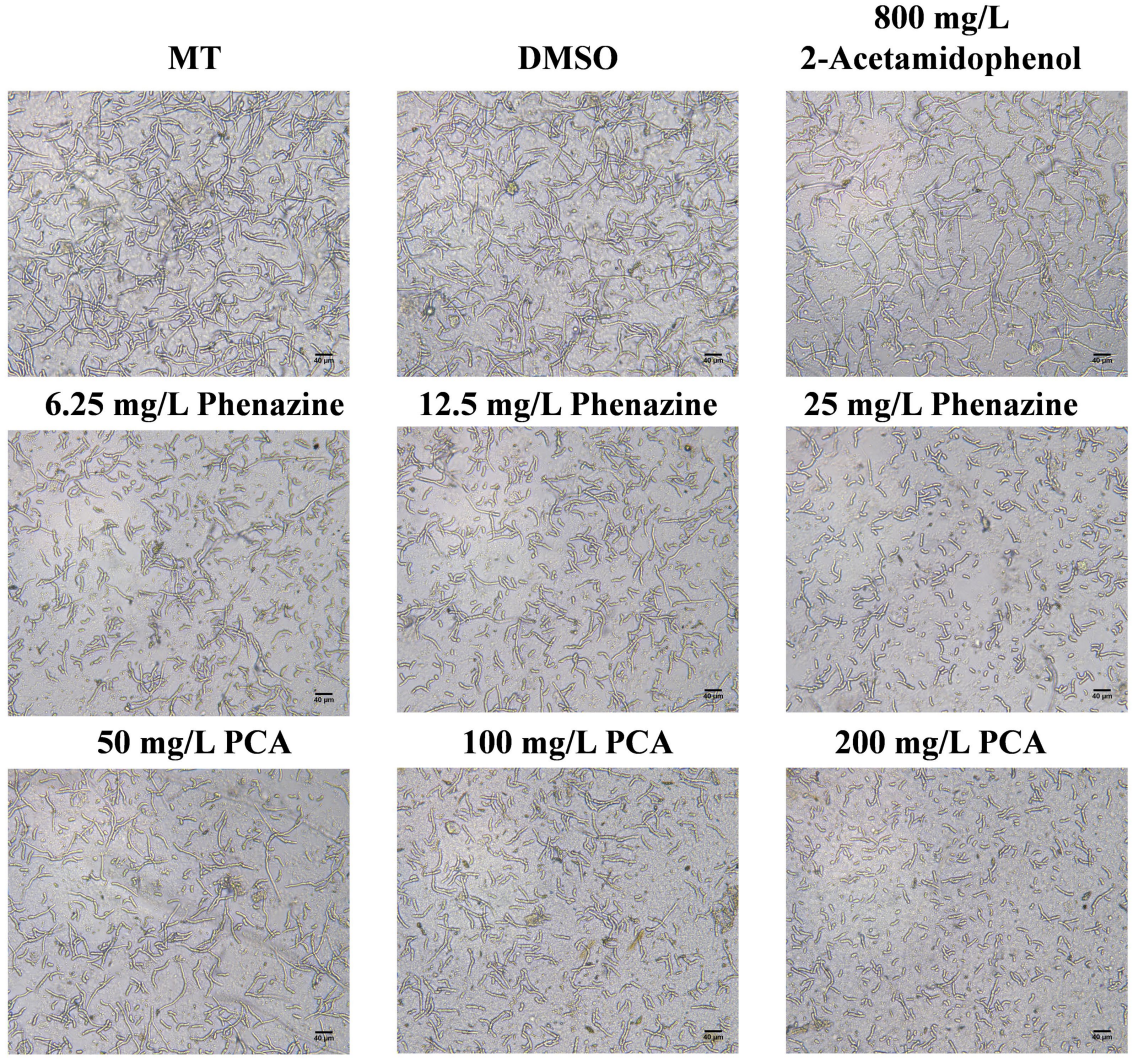
Inhibitory effect of antifungal compound phenazine, PCA and 2-acetamidophenol on spore germination of FOC TR4. We incubated 2 × 10^5^ spores/mL of FOC TR4 with MT, DMSO, 6.25, 12.5 and 25 mg/L of phenazine and 50, 100 and 200 mg/L of PCA and 800 mg/L of 2-acetamidophenol at 28°C. After 24 h incubation, images were taken under an inverted microscope. Control is 10% Dimethyl sulfoxide (DMSO) and methanol (MT).

The effects of phenazine, PCA and 2-acetamidophenol on FOC TR4 spore germination were assessed using an inverted microscope. Phenazine and PCA had a good inhibitory effect on FOC TR4 spore germination, while the inhibitory effect of 2-acetamidophenol was unstable and weak even at high concentrations. Phenazine and PCA exhibited high antifungal activity against FOC TR4 with 6.25 mg/L and 25 mg/L of the MIC. Germination rate of the FOC TR4 spore decreased to 68.55, 50.25 and 10.50% upon the addition of 6.25, 12.5 and 25 mg/L phenazine, respectively, in comparison to 96.87 and 97.15% observed when 10% DMSO and methanol (MT) was added without any compound. The FOC TR4 germination rate decreased to 71.50, 56.75 and 12.50% upon the addition of 50, 100 and 200 mg/L PCA, respectively (Figure 6). However, even when the concentration of 2-Acetamidophenol was as high as 800 mg/L, the inhibition rate of FOC TR4 was not significantly different from that of the control group. But we found that the embryonic tubule morphology of the spores was distorted and the elongation of the tubule was slightly inhibited. Previous reports have demonstrated that the impact of antifungal compounds on fungal growth is primarily dependent on the spore germination stage. Therefore, inhibiting spore germination is crucial for halting the proliferation and dissemination of fungal pathogens (Yun et al., 2020).

### Inhibitory effects of phenazine, PCA and 2-acetamidophenol on FOC TR4 mycelium growth

**Figure 7:**
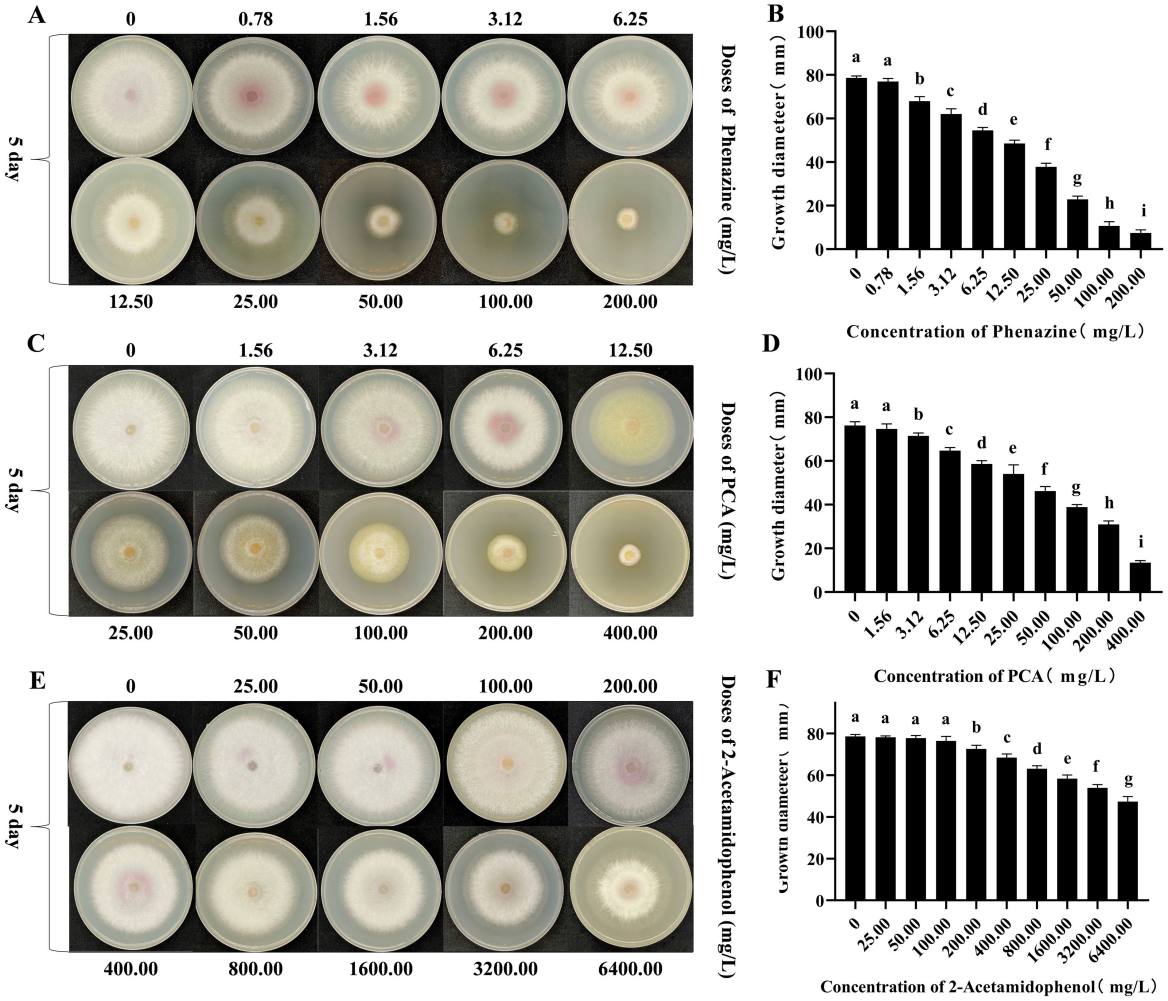
Antagonism effects of phenazine, PCA and 2-acetamidophenol against FOC TR4 *in vitro*. **(A,B)** Colony growth diameter and quantitative analysis of FOC TR4 treated with various concentrations of phenazine; **(C,D)** Colony growth diameter and quantitative analysis of FOC TR4 treated with various concentrations of PCA; **(E,F)** Colony growth diameter and quantitative analysis of FOC TR4 treated with various concentrations of 2-acetamidophenol. Different letters were used to indicate significant differences (*p* < 0.05).

Phenazine, PCA and 2-acetamidophenol exerted inhibitory effects on the mycelial growth of FOC TR4. After 5 days, phenazine and PCA demonstrated significant inhibition of FOC TR4 mycelial growth, while 2-acetamidophenol showed a less pronounced inhibitory effect. With increasing concentration, the inhibitory effect became more pronounced. Compared to the control plate (78.63 mm ± 0.86), the mycelial growth diameter was significantly reduced to 22.86 mm 2.33 in the 50.00 mg/L phenazine treatment group. At a concentration of 200.00 mg/L, phenazine significantly inhibited growth, exhibiting an inhibition rate of 90.55%. The toxic regression equation (y = 0.38x + 0.03, R^2^ = 0.98) was further established and the half-maximum median effective concentration (EC_50_) value of phenazine against FOC TR4 was 26.24 mg/L. At the same time, the concentration of PCA treatment group was 200.00 mg/L, which reduced the diameter of FOC mycelium to 30.92 ± 2.70 mm. At a concentration of 400.00 mg/L, PCA significantly inhibited the mycelial growth of FOC TR4, with an inhibition rate of 82.32%. The toxic regression equation (y = 0.27 × −0.05, R^2^ = 0.99) was further established and the EC_50_ of PCA for inhibiting FOC TR4 mycelial growth was 89.63 mg/L. However, even at concentration as high as 6400.00 mg/L, 2-acetamidophenol merely decreased the mycelial growth diameter of FOC TR4 to 47.37 ± 8.41 mm, with an inhibition rate of 39.76%. And the toxic regression equation (y = 0.14 × −0.21, R^2^ = 0.95) was further established and its EC_50_ value is as high as 11528.00 mg/L (Figure 7).

### Effects of phenazine, PCA and 2-acetamidophenol acetamide on mycelium growth and morphology of FOC TR4

**Figure 8:**
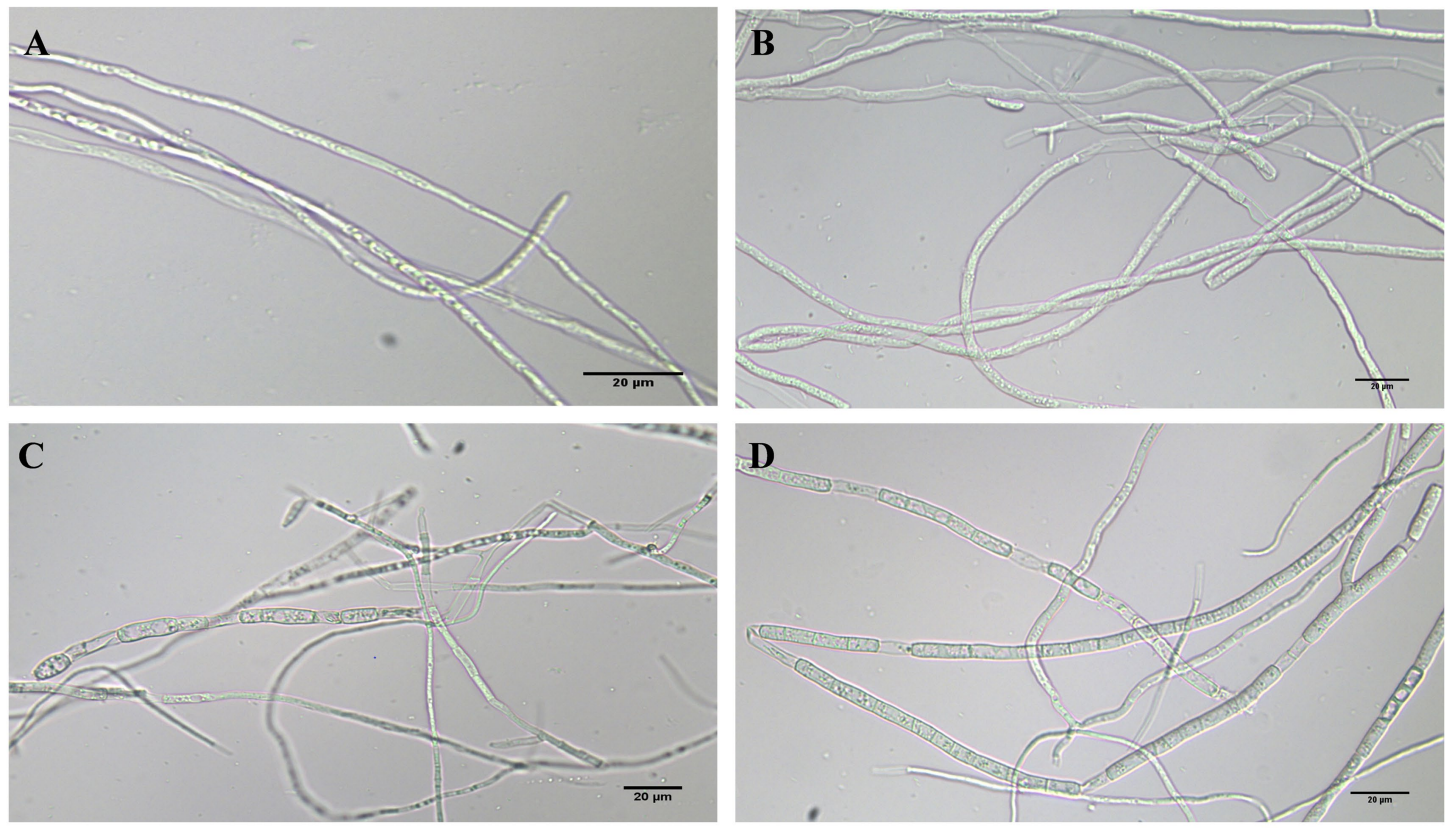
FOC TR4 and 200 mg/L phenazine, PCA and 2-acetamidophenol antagonized FOC TR4 mycelium morphology on the plate. **(A)** Normal processing group; **(B)** phenazine treatment group; **(C)** PCA treatment group; **(D)** 2-acetamidophenol treatment group.

The morphology of FOC mycelia on an antagonistic plate containing 200 mg/L of phenazine, PCA, and 2-acetamidophenol was examined using an optical microscope, with untreated pathogens serving as controls. The mycelia in the control group (CK) exhibited a smooth texture, uniform thickness, and robust growth. Phenazine primarily induced irregular expansion, entanglement, and rupture of FOC mycelia. PCA predominantly resulted in entanglement and expansion of FOC mycelia. The impact of 200 mg/L 2-acetamidophenol on FOC TR4 was relatively weak (Figure 8).

## Discussion

*Fusarium* wilt of banana is a significant fungal disease that restricts global banana production, resulting in the decline of banana production (Dita et al., 2018; Ploetz, 2006). Traditional methods of physical and chemical control frequently entail disadvantages such as environmental pollution and high expenses (Cao et al., 2022; Dadrasnia et al., 2020; Segura et al., 2021). Therefore, there exists an urgent necessity to explore novel antifungal agents characterized by eco-friendliness, efficacy, and broad-spectrum bioactivities (Xiong et al., 2017). Biological control is regarded as a novel and effective strategy for combating *Fusarium* wilt of banana, leveraging naturally existing microbial agents and their metabolites, posited to be environmentally friendly and sustainable (Ravi et al., 2022; Shafi et al., 2023). Previous studies have documented the antifungal attributes of metabolites derived from *Pseudomonas* spp., encompassing phenazine and its derivatives such as PCA, phenazine-1-methylamine (PCN), pyocyanin, 2,4-diacetylphloroglucinol, pyoluteorin, pyrrolnitrin, and lipopeptides, thereby contributing to the inhibition of hyphal growth in *Fusarium* graminearum and other plant pathogenic fungi (Gross and Loper, 2009; Shahid et al., 2018). *P. aeruginosa*, a gram-negative bacterium, is extensively distributed in soil and aquatic environments, exhibiting varying degrees of toxicity towards a broad spectrum of organisms, encompassing plants, nematodes, amoebae, insects, and mammals (Ambreetha and Balachandar, 2022; Maspoli et al., 2014; Wood et al., 2023). Nonetheless, certain strains of *P. aeruginosa* have the ability to secrete diverse metabolites antagonistic to plant pathogens, thus serving as an effective means to thwart various fungal or bacterial diseases in plants, rendering them invaluable in agricultural biotechnology (Chen et al., 2021; Shahid et al., 2021). Consequently, it can serve as a beneficial plant biocontrol bacterium for managing *Fusarium* wilt of banana.

*P. aeruginosa* has been employed as a biological agent against phytopathogenic fungi primarily attributed to its abundant production of metabolites endowed with antimicrobial activity (Abdelaziz et al., 2023; Kerr et al., 1999). In this investigation, we aimed to elucidate the putative antagonistic mechanism of *P. aeruginosa* Gxun-2 by isolating and identifying the secondary metabolites produced by this strain, and assessing their inhibitory impacts on both FOC TR4 mycelial growth and FOC TR4 spore germination. The plate diffusion assay using the ethyl acetate crude extract of Gxun-2 revealed the potential presence of antibacterial substances among the secondary metabolites produced by this strain (Figure 1). The ethyl acetate crude extract of strain Gxun-2 was subjected to purification via silica gel column chromatography, gel column chromatography, and TLC (Figure 2). Among the metabolites produced by the strain, four compounds were identified phenazine, phenazine-1-carboxylic acid (PCA), 2-acetamidophenol and aeruginaldehyde through ultraviolet, liquid chromatography and nuclear magnetic response (Figures 3–5).

Phenazine, phenazine-1-carboxylic acid (PCA), 2-acetamidophenol and aeruginaldehyde have all been reported to have antifungal activity. However, their specific biocontrol efficacy against FOC TR4 remains understudied. Prior studies have demonstrated the antifungal potential of these compounds against various fungi. For example, Phenazine produced by *P. fluorescent* had good control effect for some pathogenic fungi, such as *Rhizoctonia solani, Macrophomina phaseolina, Alternaria alternata and Sclerotium rolfsii* (Karmegham et al., 2020). The MIC values of PCA isolated from *P. fluorescens* strain 2–79 against *F. oxysporum* was 25–30 mg/L (Gurusiddaiah et al., 1986). In addition, Xu et al. discovered that the MIC of PCA derived from *Burkholderia* HQB-1 against FOC TR4 was 1.56 mg/L, which is notably lower than the outcomes of the present study. This discrepancy could be attributed to differences in strain specificity, culture conditions, or the pathogen’s sensitivity to PCA. Similarly, 2-acetamidophenol displayed moderate antibacterial activities against *Staphylococcus aureus*, *Micrococcus tetragenus*, and *P. syringae pv. actinidiae* (Psa) with MIC values of 25, 25, and 12.5 mg/L (Cui et al., 2022). Nevertheless, its antifungal effect on FOC TR4 was insignificant in our experiments, suggesting a potential specificity towards bacterial pathogens. Furthermore, Aeruginaldehyde is introduced by Ye et al. and Fakhouri et al. as a structure revision of N-mercapto-4-formylcarbostyril isolated from *P. fluorescences* and it is effective against many phytopathogenic fungi *in vitro* (Fakhouri et al., 2001; Ye et al., 2014). Thus, the fact was speculated that these compounds play a key role in the FOC TR4 antifungal activity of strain Gxun-2.

In this investigation, based on the findings from the 96-well microdilution assay, the MIC of phenazine against FOC TR4 was determined to be 6.25 mg/L, while the MIC of PCA is 25 mg/L (Figure 6). Besides, we found that phenazine and PCA can effectively inhibit FOC TR4 pore germination, and the effect of phenazine is better than that of PCA. However, the inhibitory effect of 2-acetamidophenol on FOC TR4 was not significant. On the other hand, the inhibitory effect of phenazine on FOC TR4 mycelial growth was strongest by AGAR diffusion method, followed by PCA, while the inhibitory effect of 2-acetamidophenol was not significant. The EC_50_ of phenazine against FOC TR4 mycelial growth was 26.24 mg/L, whereas the EC_50_ value of PCA inhibiting FOC TR4 mycelial growth was 89.63 mg/L. Similarly, the EC_50_ concentration of 2-acetamidophenol was also found to be 11528.00 mg/L (Figure 7). Consequently, we speculated that strain Gxun-2 inhibited FOC TR4 mainly through the production of phenazine and PCA, and they could effectively inhibit the normal growth of FOC TR4 and inhibit its spore germination. Compared with PCA, phenazine exhibited more pronounced antibacterial activity. However, the inhibitory effect of 2-acetamidophenol on FOC TR4 was not significant, and the inhibitory activity of aeruginaldehyde against FOC TR4 remained to be explored. The observed variations in MIC and EC_50_ values between our study and previous reports can be attributed to several key factors, including experimental conditions (such as temperature, pH, and incubation duration), the varying sensitivity of pathogens to antifungal compounds, and differences in experimental methodologies (pertaining to media selection, and initial spore concentration).

Utilizing electron microscopy and fluorescent techniques, P852, a novel cyclic peptide isolated from *B. amyloliquefaciens* L-H15, treatment caused the morphological change of *F. oxysporum* cells and disrupted its cell structure, including formation of blebs, broken hyphae, deformation of membrane, intracellular organization disruption, pore formation, and cell lysis (Han et al., 2019). Additionally, the crude extracts of *Chaetomium globosum* LB-2 potently inhibited the *F. oxysporum* mycelia growth, resulting in swelling and atrophy of the mycelia (Liu and Chang, 2018). In our study, mycelia in the group treated with FOC TR4 and strain Gxun-2 extracts displayed distortions, thickening, increased branching, disordered growth, curvature, and knotting (Figure 1D). Similarly, microscopic examination revealed that phenazine and PCA induced significant alterations in the morphology of FOC TR4 mycelium, characterized by folding, bending, and fracturing, concurrently reducing spore production (Figure 8). These findings corroborate previous research, which highlighted the impact of strain Gxun-2 on FOC TR4 mycelia, and further transcriptomic analyses indicated disruptions in membrane and cell wall integrity, as well as perturbations in peroxidase synthesis under Gxun-2 stress (Li et al., 2022). Therefore, we contend that phenazine and PCA are key compounds that cause the distortion of FOC TR4 mycelium morphology and may interfere with the formation of membranes and cell walls of FOC TR4. Additionally, Niphimycin C, isolated from *Streptomyces yongxingensis* sp. nov. (JCM 34965), demonstrated potent antifungal effects against FOC TR4, inhibiting mycelial growth and spore germination. It disrupted mitochondrial function and metabolism in FOC TR4 cells, diminishing key enzyme activities within the tricarboxylic acid (TCA) cycle and electron transport chain (ETC) (Chen et al., 2022). Collectively, these observations underscore the possibility that antimicrobial compounds may harness multiple mechanisms of action to inhibit FOC TR4 growth, including the disruption and hindrance of essential metabolic pathways. However, the specific inhibition of phenazine and PCA on FOC TR4 still needs to be further explored. These studies will pave the way for the development of highly effective fungicides to improve the efficacy of agricultural applications.

## Conclusion

*P. aeruginosa* Gxun-2 extracts had a strong antifungal activity against FOC TR4 and its extracts effectively inhibited the mycelial growth and spore germination of FOC TR4. Among the metabolites produced by the strain Gxun-2, four compounds were identified phenazine, phenazine-1-carboxylic acid (PCA), 2-acetamidophenol and aeruginaldehyde through liquid chromatography and nuclear magnetic response. Among these, phenazine and PCA demonstrated the most notable inhibitory activity of FOC TR4. Phenazine exhibited high antifungal activity against FOC TR4 with 6.25 mg/L of the MIC. The EC_50_ was determined to be 26.24 mg/L utilizing the toxicity regression equation. PCA exhibited against FOC TR4 with 25 mg/L of MIC and 89.63 mg/L of EC_50_. Morphological observations revealed, Phenazine and PCA triggered substantial morphological transformations in the mycelia of FOC TR4, encompassing folding, bending, fracturing, and diminished spore formation. This study showed that *P. aeruginosa* Gxun-2 had the potential as a biocontrol agent for preventing *Fusarium* wilt of banana from the infection of FOC TR4 and it inhibited FOC TR4 mainly by producing phenazine and PCA, and phenazine had the best inhibitory effect.

## Data Availability

The original contributions presented in the study are included in the article/supplementary material, further inquiries can be directed to the corresponding authors.
